# Lafora Disease during a Seven-Year Period, Bosnian and Herzegovinian experience

**Published:** 2019

**Authors:** Edin BEGIC, Haris BRADARIC, Zijo BEGIC, Amra DOBRACA

**Affiliations:** 1 Health Care Centre, Maglaj, Bosnia and Herzegovina; 2 Department of Pharmacology, School of Medicine, Sarajevo School of Science and Technology, Sarajevo, Bosnia and Herzegovina; 3 Department of Cardiology, Pediatric Clinic, Clinical Center University of Sarajevo, Sarajevo, Bosnia and Herzegovina; 4 PhD Student, Faculty of Medicine, University of Tuzla, Tuzla, Bosnia and Herzegovina

**Keywords:** Progressive myoclonus epilepsy, Lafora disease, NHLRC1 mutation

## Abstract

Lafora progressive myoclonus epilepsy (Lafora disease, LD) is a fatal autosomal recessive neurodegenerative disorder (with an onset in teenage years in previously normal adolescents). This paper represents a view of a patient diagnosed with Lafora progressive myoclonus epilepsy, over a course of seven years. A description of the initial manifestation of symptoms, doctors' attempts to combat the symptoms with drug treatment, further attempts towards reaching the correct diagnosis, the final confirmation of the Lafora diagnosis (mutation in the *NHLRC1* gene), and the current state of the patient is presented. The absence of a positive family history, the lack of staff specialized in dealing with this or similar pathology, and the diagnostic inability to characterize this type of disorder in Bosnia and Herzegovina have led to a fair delay in diagnosing and beginning of an adequate pharmacological treatment. Overall, recent identification of LD cases in Bosnia and Herzegovina warrants an establishment of a Centre for Genetic Testing in order to ensure more humane counseling of an entire family whose family member(s) might be diagnosed with this devastating and currently an incurable disorder.

## Introduction

This paper represents a view of a patient diagnosed with Lafora progressive myoclonus epilepsy (Lafora disease, LD), over a seven-year-long period, including the beginning of symptoms, the attempts to combat the symptoms with drug treatment, the attempts at reaching the correct diagnosis, the confirmation of the diagnosis (mutation in the *NHLRC1* gene), and the current state of the patient. Parents as legal representative signed informed consent. Local ethical approval for case presentation was obtained.

## Case report

In Apr 2008, a 9-yr-old girl with initials A.S. (born in 1999, in Maglaj, Bosnia and Herzegovina) was admitted into the General Hospital Tesanj (Tesanj, Bosnia and Herzegovina) with a severe headache, mental confusion, high fever, and a cough. Neither the patient's own medical history nor that of her family contained details of any specific disorder. Both her birth weight (3850 gr) and her birth length (58 cm) were considered normal. Prior to that admission to the hospital, she manifested no cognitive disabilities. She was diagnosed with having epilepsy with mental disorientation and she was treated with phenobarbitone, sodium valproate, and lamotrigine. Then, in 2010, she started having seizures: they would begin with nausea, impaired vision, and a loss of consciousness. During the seizures, her eyes were tightly closed and she exhibited no twitching of her legs nor her arms. After ten to thirty min, the patient would regain consciousness and would complain of feeling cold at the terminal part of extremities. Additionally, she would feel pain in her ankles, with bruising joints, after which the pain would progress towards her toes, followed by swelling and reddening. Magnetic resonance imaging (MRI) of the patient's head showed no apparent changes in the structure of the patient's brain. 

During the third year following the onset of her symptoms and her first seizure, the patient experienced a different type of "seizure" during her sports class: she began to walk aimlessly and insecurely, with tottering, she had a fixed gaze, pale face, and was rambling (calling a friend by her name over and over again). However, she did not lose her balance and did not fall. In addition, she manifested no twitching of extremities. That particular "seizure" lasted for about ten minutes, after which she regained consciousness. However, following that particular seizure the patient failed to re-establish her previous state: she had absent gaze, difficulty using cutlery, she lost her sense in space and time, and she did not recognize her parents and people she previously knew around her. On one occasion, she had a generalized epileptic seizure.

In July 2011, the patient experienced a sudden loss of consciousness, and she lost her balance and fell, her body becoming rigid and cyanotic, without twitching of the extremities, while her eyes were closed. At the time, the patient's pupils were round, symmetrical, with proper response to light. Following recovery from this seizure, the patient had difficulty walking independently; she walked aided and her walk was atactic (suggesting a lack of muscle coordination), with significant deviation to the left. She also had difficulty in performing a walk on heels and toes and could not walk in a straight line. Dystonia of torso and extremities was present. Romberg was unstable, with a deviation to the left. Finger to nose test was abnormal (suggesting dysmetria). Gross motor abilities test was in order. Muscle tendon reflex (MTR) was symmetrical, live. Right, Babinski sign was positive. Lumbar puncture test, as well as findings of ophthalmologists and cardiologists, were without noticeable pathologies. Hormonal status of the patient was within the normal range. An electroencephalogram test (EEG) showed continuously discharging of the high voltage Delta Theta activity with interposed sharp and steep waves of high voltage and paroxysmal high voltage spikes, as well as multiple spike-and-wave discharges. During the stay at the hospital, the EEG registration did not change. The patient was subjected to additional MRI scans which revealed no apparent changes in the brain structure. Seizures were classified as visually partial twitches, eventually becoming myoclonic, and generalized tonic-clonic twitching. At this time, the patient exhibited evident cognitive deterioration. No cranial nerve deficits were present, but rapid irregular myoclonic twitches that were palpable at rest were present and were increased by voluntary movements of the upper limbs. There was no sign of Babinski. The patient was able to cooperate with the doctor during the examination. In addition, the patient was decelerated with a little verbal and motor activity.

Genetic testing was carried out in Genoa, Italy, on Mar 2013, from the DNA that was extracted from leukocytes of peripheral blood. Analysis of exons 1-4 of the *EPM2A *gene* (NM_005670)* and the single exon, exon 1, of the *NHLRC1 *gene* (NM_198586)*, using direct sequencing, was performed. Multiplex Ligation-dependent Probe Amplification (MLPA) method was used to examine the genes and the genomic region that includes the region from exons 1 and 4 of the *EPM2A* gene and exon 1 of the *NHLRC1* gene. The genetic testing identified that the patient is the carrier of the homozygous 1-bp deletion, 990G, within the *NHLRC1 *gene. Following the result of the genetic testing, clonazepam was included in the patient's therapy. 

In July 2015, levetiracetam was added to the therapy. In May 2015, treacheostoma was placed, as well as percutaneous endoscopic gastrostomy. Currently, (Apr 2017), the patient is bedridden, in a continuous horizontal position, with the rapid irregular myoclonic twitches noticeable at rest, and increased by voluntary movements of the upper limbs. Furthermore, the patient also experiences frequent episodes of fever, which is insensitive to antipyretics.

## Discussion

Lafora progressive myoclonus epilepsy is a fatal autosomal recessive neurodegenerative disorder (with an onset in teenage years in previously normal adolescents (1), although there are reports of cases of the illness being diagnosed in patients in their mid-twenties (2)) characterized by the presence of glycogen-like intracellular inclusions called Lafora bodies (majority of patients carry mutations in either the *EPM2A* or *EPM2B *(also termed *NHLRC1 *gene) genes, encoding laforin, a glucan phosphatase, and malin, an E3 ubiquitin ligase, respectively) (3). 

In LD, gene carriers are asymptomatic, and in family history, there are usually no examples of any disease with similar symptoms. It is relatively common in the Mediterranean part of Spain, France, and Italy, in restricted regions of central Asia, India, Pakistan, northern Africa, and the Middle East, in ethnic isolates from the southern United States and Quebec (4). In addition to the case of LD described herein, two sibling cases of LD were registered in Bosnia and Herzegovina in 2006 and 2007. In general, diagnosis of LD is based on clinical examination, the EEG findings, as well as genetic testing of two genes that are associated with LD: *EPM2A* or* EPM2B *(*NHLRC1*)*.* The diagnosis of LD in developing countries could be made by skin and axillary sweat gland biopsy (eccrine or apocrine) by periodic acid-Schiff stain which is more affordable (detection of Lafora bodies). Lafora bodies can also be detected in the brain, liver, skin, or muscle tissue, anterior pituitary, hypothalamus, and pancreas (5, 6). LD is characterized by fragmentary, symmetric, or generalized myoclonus and/or generalized tonic-clonic seizures, visual hallucinations (occipital seizures), and progressive neurologic degeneration, including cognitive and/or behavioral deterioration, dysarthria, and ataxia beginning in previously healthy adolescents (4).

 In the case presented herein, despite of three-year long wandering in the establishing of the correct diagnosis, the initial success in the treatment was nevertheless achieved by pharmacological treatment of the patient's disease symptoms. With the progression of the disease, as time passed, the success of the therapy was at a diminished level. The absence of a positive family history, the lack of staff specialized in dealing with this or similar pathology, and the diagnostic inability to characterize this type of diagnosis in Bosnia and Herzegovina have led to a fair delay in diagnosing and beginning of the adequate pharmacological treatment. Currently, there is no specific treatment for LD; however, commonly used antiepileptic therapy (palliative treatment) for the management of myoclonus may improve symptoms in the early stages of the disease (combinations that include valproate, phenobarbital, benzodiazepines, piracetam, levetiracetam, and zonisamide are useful for symptomatic treatment) (7,8). 

## Conclusion

LD is a rare progressive myoclonus epilepsy, with a very uncertain and poor prognosis, and only treatment of symptoms can be carried out, and it requires a multidisciplinary approach in the treatment and understanding of the overall community. Recent identification of LD cases in Bosnia and Herzegovina warrants an establishment of a Centre for Genetic Testing in order to ensure more humane counseling of an entire family whose family member(s) might be diagnosed with this devastating and currently an incurable disorder.
